# Supplementary figures and images for: Checkpoints of apicomplexan cell division identified in Toxoplasma gondii
Source: PLoS Pathog. 2017 Jul 3;13(7):e1006483. doi: 10.1371/journal.ppat.1006483 (PMC5510908; doi:10.1371/journal.ppat.1006483)

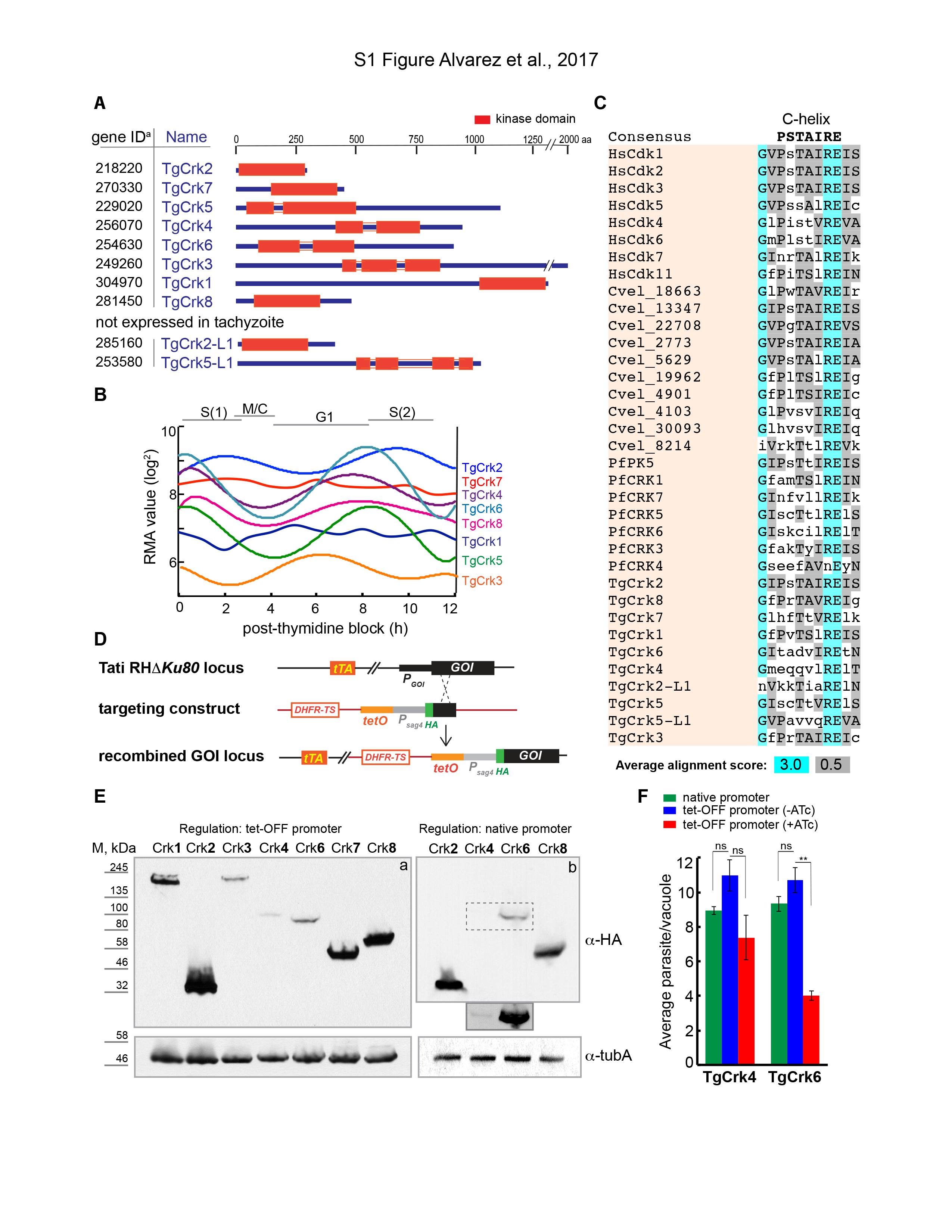

Supplement: S1 Fig — (A) Diagram shows (a) TGME49_ gene ID number, suggested name, relative length and domain structure of T. gondii Crks. The conserved protein kinase domain (red block) of a few TgCrks contains one or more inserts shown as breaks in the structure. (B) The graph shows mRNA dynamics of TgCrks expressed during the tachyzoite cell cycle (toxoDB). Polynomial trend curves were applied where possible. (C) Alignment of the C-helixes of T. gondii, P. falciparum, C. velia Crks and selected H. sapiens Cdks revealed high divergence in the sequences responsible for binding cyclins. Note that with exception of TgCrk5-L1, T. gondii Crks retained conservative threonine in the T-loop that becomes phosphorylated upon binding of a cognitive cyclin. (D) Schematics of recombination event establishing a tet-OFF model. Targeting construct contains 3’ fragment of the gene of interest (GOI) and is cloned in-frame with 3xHA epitope tag under Sag4 promoter fused with tetracycline operators (tetO). A recombination event replaces the GOI promoter with a tetracycline-regulatable (tet-OFF) promoter in a parent expressing a tetracycline transactivator (tTA). (E) Comparative expression of HA-tagged TgCrks in corresponding tet-OFF mutants (image a) and in transgenic strains expressing endogenously tagged TgCrks (image b). To enhance TgCrk4HA signal, a section of the image b (dotted box) was overexposed and is shown below. The total lysates of the transgenic parasites grown under permissive conditions for 30–32 h (tet-OFF mutants) were analyzed by Western blotting with α-HA antibody. Loading control was provided by probing samples with α-TubulinA antibody. Note that under alternative regulation (tet-OFF versus native promoter) protein expression levels of the TgCrks remained relatively similar and they nearly matched the steady-state levels of the corresponding mRNAs shown in the panel B, suggesting a minimal role of the transcription initiation. (F) Division rate of TgCrk4 and TgCrk6 tet-OFF mutants we [file ppat.1006483.s002.tif]

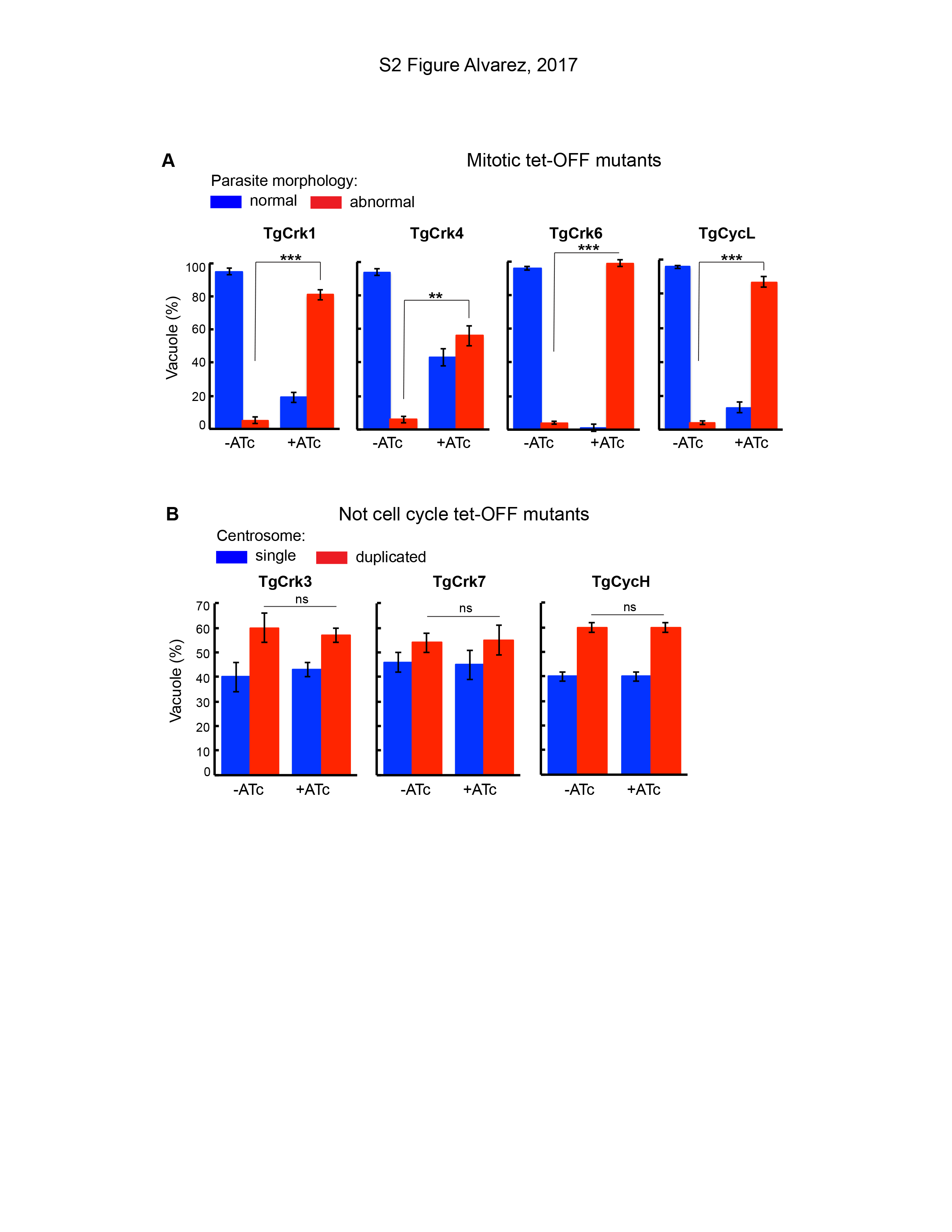

Supplement: S2 Fig — (A) Asynchronous population of the putative mitotic tet-OFF mutants grown for 24 h with and without 1μg/ml ATc were analyzed by IFA using α-IMC1, α-Centrin1 and DAPI staining. The following deficiencies were quantified: TgCrk1 and TgCycL—severely mis-shaped parasites and the loss of uniform IMC1 distribution; TgCrk4—odd number of parasites/vacuole, asynchronous vacuoles, and improper centrosome/parasite ratio; TgCrk6 –odd number of parasites/vacuole, severe DNA mis-segregation. The average of 25–50 vacuoles was evaluated at each condition and statistical difference in the number of deficient vacuoles is indicated with asterisks (*** p ≤ 0.0001, ** p ≤ 0.001. (B) Single (blue) and duplicated (red) centrosomes (α-Centrin1) in 100 randomly picked vacuoles were quantified in asynchronous populations of the TgCrk3, TgCrk7 and TgCycH tet-OFF mutants grown for 24 h with and without 1μg/ml ATc. Nucleus and parasite surface were visualized with DAPI and α-IMC1 staining. Downregulation of the analyzed factors did not significantly affect the relative length of G1 (single centrosome) and S/M/C (duplicated centrosome) phases or parasite morphology and DNA segregation (ns—not significant). (TIF) [file ppat.1006483.s003.tif]

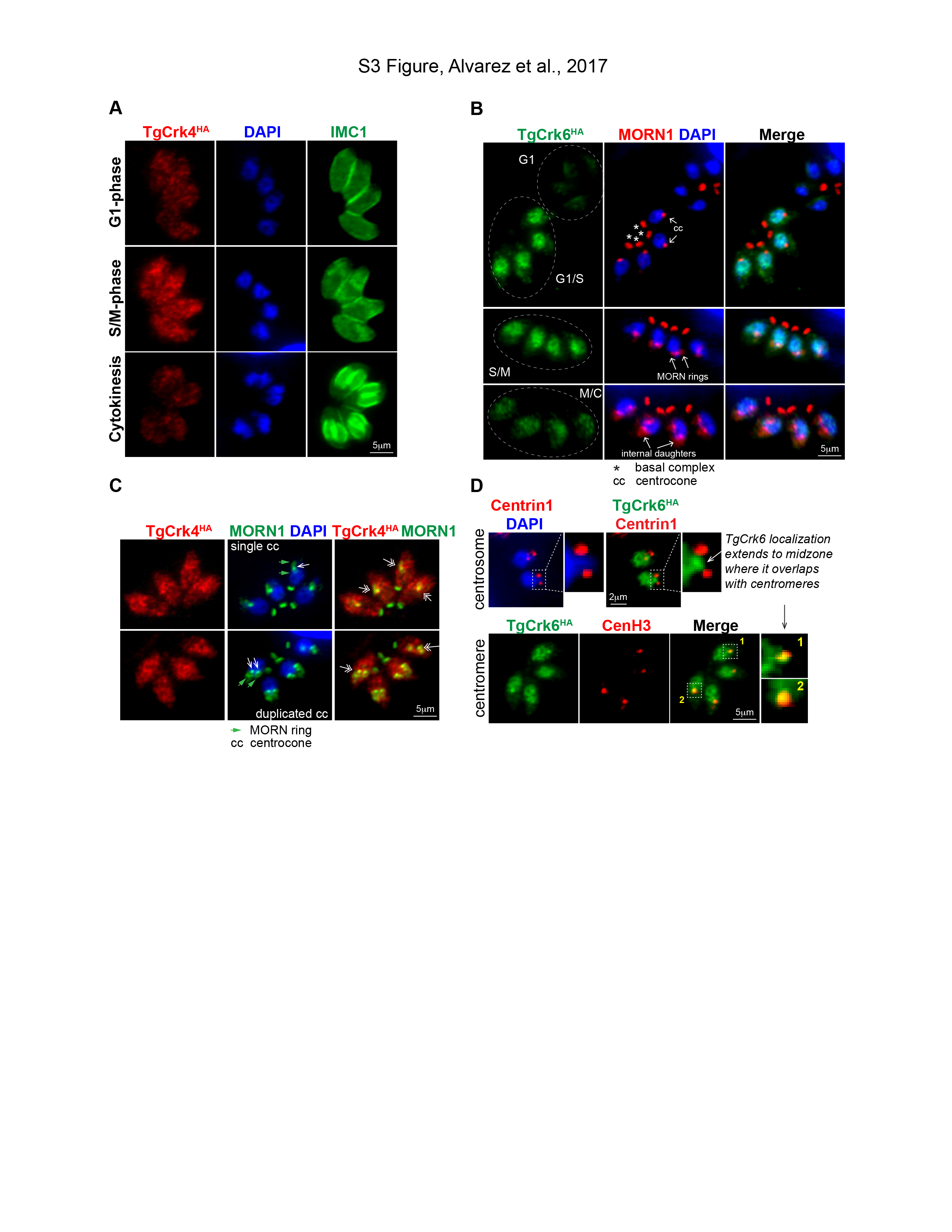

Supplement: S3 Fig — (A) Expression of TgCrk4HA kinase under native regulation was analyzed using α-HA (red), α-IMC1 (green) antibody and nuclear DAPI staining (blue). Cell cycle phases were determined based on intensity of the nuclear staining and progression of internal budding. IFA established the peak of TgCrk4HA expression is in S/M phase. (B) Endogenously tagged TgCrk6HA had peak expression during centrocone expansion and duplication in G1/S and S/M phases. The morphological transitions of MORN1 protein (red), the shape of the nucleus and intensity of DNA staining (DAPI, blue) were used to determine specific cell cycle phases. TgCrk6HA first appeared in late G1 when the centrocone is expanded (G1/S vacuole), maintained maximum expression during S-phase and mitosis (S/M vacuole) until the spindle breaks in post-anaphase (M/C vacuole) and the duplicated centrocone compartment started to recede. Dotted line shows vacuole boundary. (C) Endogenously tagged TgCrk4HA is expressed in the cytoplasm. Co-staining with centrocone marker MORN1 (green) confirmed peak of TgCrk4HA expression during centrocone enlargement and break in S/M phase prior karyokinesis. Co-staining with DAPI (blue) showed perinuclear accumulation of TgCrk4HA at the sites of active mitosis. TgCrk4HA localization near the centrocone prior to (upper panel) and after duplication (lower panel) is indicated with double-headed arrows. (D) TgCrk6HA (green) accumulation in the nuclear extension between duplicated centrosomes revealed by co-staining with α-Centrin1 antibody (red) and DNA staining with DAPI (blue, upper panel). Transient increase in the centromere/spindle region was confirmed by co-localization with centromere protein CenH3 (α-CenH3, red, lower panel). (TIF) [file ppat.1006483.s004.tif]

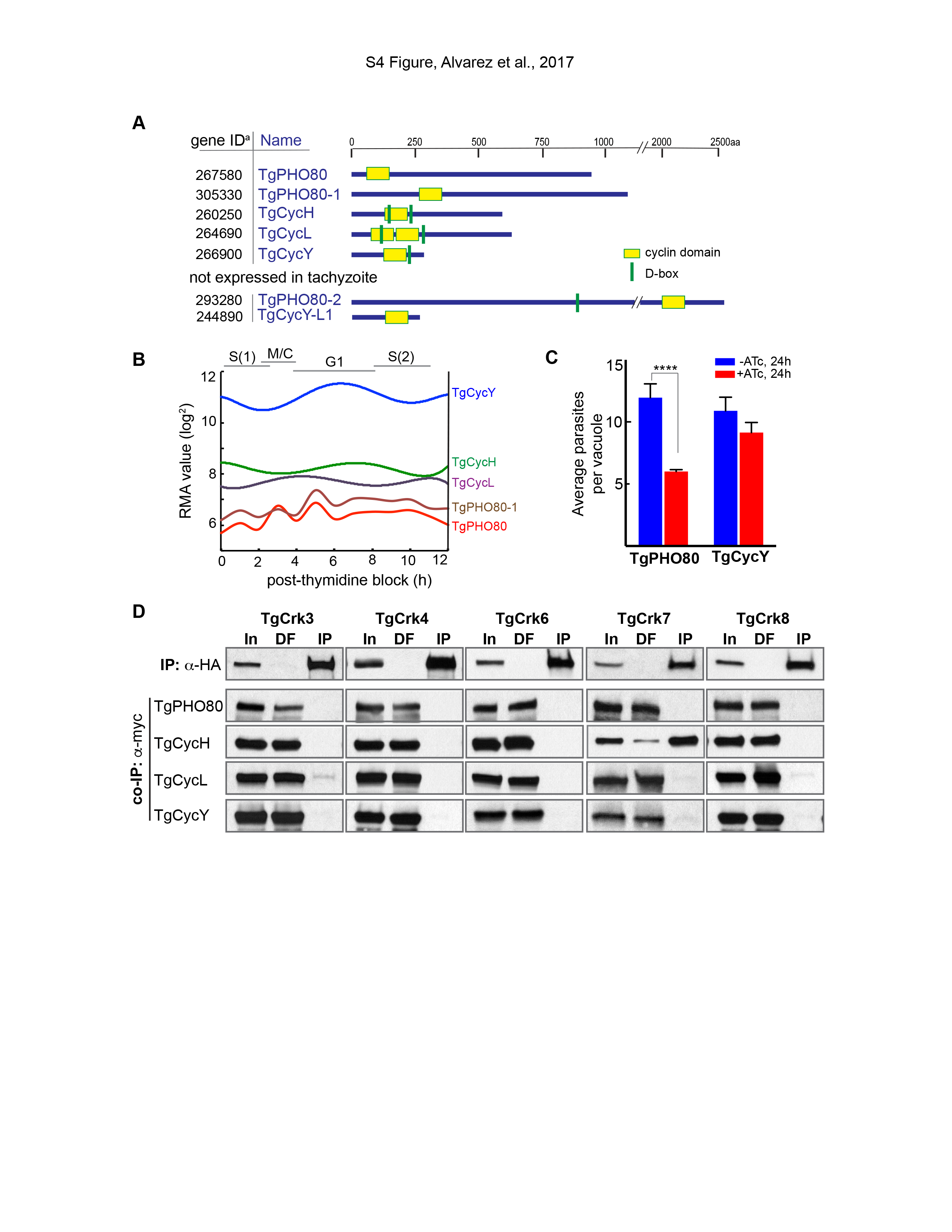

Supplement: S4 Fig — (A) Diagram shows (a) TGME49_ gene ID number, suggested name, relative length and position of the cyclin domain (yellow box) and D-box (green vertical line) in the seven cyclin-like proteins identified in T. gondii genome. To predict D-boxes, protein sequences were analyzed by GPS-ARM software and only those with a score 4.5 or higher are shown. T. gondii possesses three P-cyclins with TgPHO80-2 mRNA up regulated in bradyzoite transcriptomes (toxoDB). Due to complexity of the genomic locus, TgPHO80-1 was not studied here. (B) The graph shows cell cycle profiles of mRNA expression (dotted lines) of the putative cyclins (toxoDB). Polynomal trend curves (solid lines) were applied where possible. (C) Division rates of TgPHO80 and TgCycY tet-OFF mutants were evaluated after 24 hours growth in the presence (red) or absence (blue) of 1μg/ml ATc. The parasite average per 50 vacuoles was quantified in 3 biological replicates and shown on the graph. Multiple comparisons performed via 2-way ANOVA with Bonferroni correction. Error bars represent the standard deviation of each sample and significant difference (P<0.05) indicated by asterisks. (D) Interaction of TgCrk3, TgCrk4, TgCrk6, TgCrk7 and TgCrk8 with TgCyclins were examined in parasites co-expressing endogenously tagged TgCrksHA and ectopic copes of TgCyclins tagged with 3xmyc-epitope (list if used transgenic strains is in the S1 Table). The soluble fraction before [In] and after immunoprecipitation [DF], and the protein complexes on the beads [IP] were probed with α-myc antibody to detect TgCyclins and with α-HA to verify pulldown of TgCrks (top panel). The results confirmed conservative interaction between TgCrk7 and TgCycH, while no significant complexes were detected between kinases TgCrk3, TgCrk4, TgCrk6 and TgCrk8, and any of the analyzed TgCyclins. (TIF) [file ppat.1006483.s005.tif]

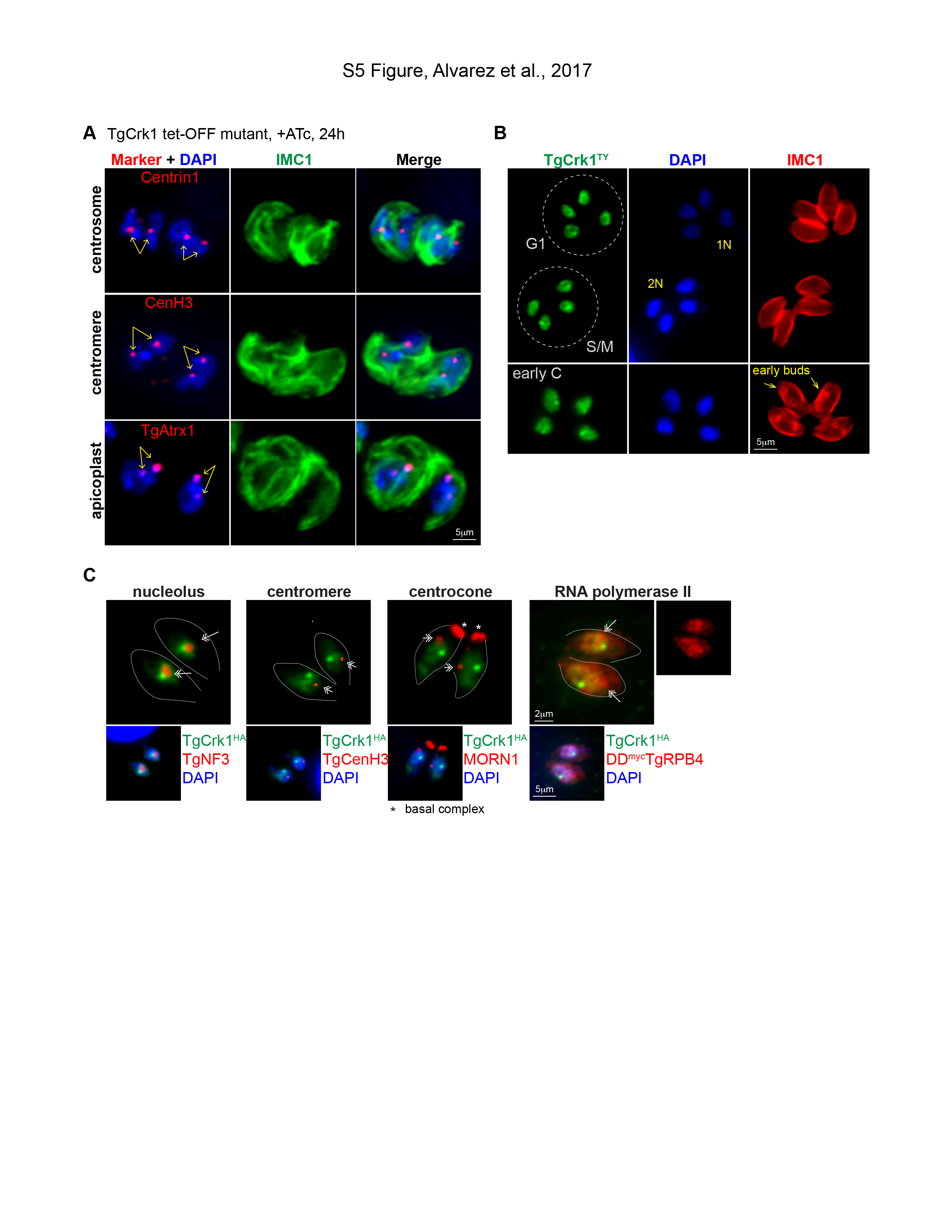

Supplement: S5 Fig — (A) TgCrk1 tet-OFF mutant parasites were grown for 24 h with 1μg/ml ATc and co-stained with antibodies against the surface marker IMC1 (green), nuclear DNA dye DAPI (blue) and markers for the centrosome (α-Centrin1), centromere (α-CenH3) and apicoplast (α-Atrx1). TgCrk1-deficiency did not affected duplication and segregation of the major organelles and structures essential for parasite survival. (B) IFA analysis of TgCrk1TY cell cycle expression. Endogenously tagged TgCrk1Ty was visualized with α-Ty antibody, and co-stained with α-IMC1 to detect internal buds (M/C stage) and DAPI stain to discriminate replicated DNA (G1 versus S/M/C stages). No significant cell cycle dynamics was observed in TgCrk1 expression. In a similar analysis we found that TgCrk3Ty, TgCrk7HA and TgCrk8HA were also constitutively expressed in tachyzoites. (C) Co-localization studies of TgCrk1HA (α-HA) with markers of the known nuclear compartments: nucleolus (α-TgNF3), centromere compartment (α-TgCenH3) and centrocone (α-MORN1). TgCrk1 is distantly related to Cdk11 kinase known to regulate gene expression and splicing in higher eukaryotes. We co-expressed TgCrk1HA and a subunit of the DNA-dependent RNA polymerase II complex, DDmycTgRBP4 (α-myc), which was localized to nascent particles concentrated in multiple nuclear foci of the tachyzoite. Like the other nuclear sub-compartments examined, the DDmycTgRBP4 structures were also not associated with the nuclear structure defined by the TgCrk1-TgCycL complex. Nuclear compartments and a major cluster of the nascent particles (TgRPB4) are indicated with arrows. Parasites are outlined with gray. The small image on the bottom shows location of each compartment in the nucleus (DAPI, blue). (TIF) [file ppat.1006483.s006.tif]
